# Conceptual evolution of 3D printing in orthopedic surgery and traumatology: from “do it yourself” to “point of care manufacturing”

**DOI:** 10.1186/s12891-021-04224-6

**Published:** 2021-04-16

**Authors:** Jose Antonio Calvo-Haro, Javier Pascau, Lydia Mediavilla-Santos, Pablo Sanz-Ruiz, Coral Sánchez-Pérez, Javier Vaquero-Martín, Rubén Perez-Mañanes

**Affiliations:** 1 Orthopaedic Surgery and Traumatology Department, Hospital General Universitario Gregorio Marañón, Calle Doctor Esquerdo, 46, Postal code, 28007 Madrid, Spain; 2 Advanced Planning and 3D 1Manufacturing Unit, Hospital General Universitario Gregorio Marañón, Madrid, Spain; 3 Faculty of Medicine, Department of Surgery, Universidad Complutense, Madrid, Spain; 4 Instituto de Investigación Sanitaria Gregorio Marañón, Madrid, Spain; 5 Departamento de Bioingeniería e Ingeniería Aeroespacial, Universidad Carlos III de Madrid, Madrid, Spain

**Keywords:** 3D printing, Manufacturing university hospital, POC manufacturing, Preoperative planning, Biomodels, Surgical guides, Custom implants

## Abstract

**Background:**

3D printing technology in hospitals facilitates production models such as point-of-care manufacturing. Orthopedic Surgery and Traumatology is the specialty that can most benefit from the advantages of these tools. The purpose of this study is to present the results of the integration of 3D printing technology in a Department of Orthopedic Surgery and Traumatology and to identify the productive model of the point-of-care manufacturing as a paradigm of personalized medicine.

**Methods:**

Observational, descriptive, retrospective and monocentric study of a total of 623 additive manufacturing processes carried out in a Department of Orthopedic Surgery and Traumatology from November 2015 to March 2020. Variables such as product type, utility, time or materials for manufacture were analyzed.

**Results:**

The areas of expertise that have performed more processes are Traumatology, Reconstructive and Orthopedic Oncology. Pre-operative planning is their primary use. Working and 3D printing hours, as well as the amount of 3D printing material used, vary according to the type of product or material delivered to perform the process. The most commonly used 3D printing material for manufacturing is polylactic acid, although biocompatible resin has been used to produce surgical guides. In addition, the hospital has worked on the co-design of customized implants with manufacturing companies.

**Conclusions:**

The integration of 3D printing in a Department of Orthopedic Surgery and Traumatology allows identifying the conceptual evolution from “Do-It-Yourself” to “POC manufacturing”.

## Background

3D printing has emerged as a disruptive technology in Orthopedic Surgery and Traumatology [1, 2]. In this area, it has been used to create customized biomodels (replicas of patient anatomy), devices, instruments and implants, to plan and simulate complex surgical procedures, or as a teaching or communication tool [3]. The increase in accessibility of this technology has moved hospitals towards the implementation of their own in-house 3D printing programs, intending to create knowledge internally and to reduce both delivery times and costs of the 3D printed models [4]. In this scenario, a *manufacturing university hospital* may act as a hub that may combine in-house 3D printing with distributed production. This idea arises not to compete against the traditional medical industry, but to generate value in personalized medicine. There are several references to this approach, where professional teams with the necessary resources have developed knowledge based on their individual experience, allowing a qualitative leap in patient-centered medicine [5–7].

The integration of this technology within the hospitals started with a “Do it yourself” approach, where realistic reproductions were 3D printed by innovative individuals at a minimum cost. These initial steps have now led to new production models in which the manufacturing hospitals are identified as network hubs, reconciling in-house manufacturing and outsourcing. This solution strengthens collaborative work, ensures that hospital manufacturing becomes the standard of care, and efficiently adjust available resources to the existing limitations [5, 8]. This change of paradigm frees hospitals from the restrictions imposed by the commercial catalog, allowing them to propose, manufacture, evaluate and participate in emerging lines such as custom-made implants manufacturing [9–12] or tissue bio-printing [13–15].

New production models such as “point-of-care (POC) manufacturing” provide for this, making it possible to respond to space needs or expensive installations, to bring together the technical competence profile in industrial aspects, or to keep its portfolio of services up to date without depending on the obsolescence of the machines and manufacturing materials, which is very rapid for this type of technology [5].

The purpose of this study is to present the results of the integration of 3D printing technology in a Department of Orthopedic Surgery and Traumatology and to identify the POC productive model as a paradigm of personalized medicine, allowing to conciliate indication and surgical planning with the design and manufacture of specific patient solutions.

## Methods

In order to define the possibilities of integrating 3D printing technology in a Department of Orthopedic Surgery and Traumatology, we present an observational, descriptive, retrospective, and monocentric study which includes the additive manufacturing processes carried out in the Department at Hospital General Universitario Gregorio Marañón (Madrid. Spain). The time interval starts from the creation of the Advanced Planning and 3D Manufacturing Unit (UPAM3D) in November 2015 to March 2020, date on which the UPAM3D projects were temporarily interrupted as a result of the COVID-19 pandemic.

**Fig. 1:**
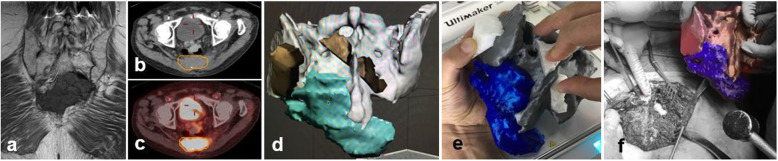
Sacral tumor. Workflow. Patient radiological images **a-c**, 3D digital model **d**, Products [3D printed biomodel and Surgical guides] **e**, Product utility [Intraoperative utility] **f**

An additive manufacturing process allows transforming a digital model into a real and tangible three-dimensional product. 3D digital models can be obtained from different sources: digital radiological studies, three-dimensional scanning, computational design (CAD) or reverse engineering. When the digital model is ready, the products are built layer by layer, using different technologies and materials depending on the final application [16, 17]. Figure 1 summarizes the 3DP workflow in a patient with a sacral tumor, enabling the fabrication of the biomodel and surgical guides for tumor resection.

In this study, we have defined the following activity variables to analyze the additive manufacturing process:

- Area of expertise: it identifies the specific area in the department that originated the activit. Possible values are Traumatology, Upper Limb, Spine, Foot and Ankle, Pediatric orthopedic, Reconstructive, and Oncology, Basic research, and some specific research lines (3DP research, University collaborative projects).
- Required product: The products can be 3D printed biomodel, surgical guide/instrument for an interventional procedure, or an instrument for surgical navigation.
- Product utility: This variable takes into account the primary utility of the product. Although these products have great value as a communication tool in most cases, it has been described as such only when communication was the essential utility of the product. Other utilities have been preoperative planning, intraoperative utility, instrumental or research.
- Material delivered to perform the process: this identifies the original information provided by the requesting user. It may be a medical record number or radiological images in DICOM files [MRN/DICOM], a 3D digital model design [3D model], or other materials such as a drawing or a tool with design improvement suggestions, which need to be reverse-engineered to obtain a 3D Model.
- Work time: Time allocated by the facultative team and technical team to develop the project, which includes obtaining and designing the 3D model, preparing it for the printing process, and post-processing the product after manufacturing before delivery.
- 3D Printing time: Time required by the 3D printer to manufacture the product.
- Quantity of 3D Printing material: Quantity of material (in grams) used by the printer for each of the products obtained.
- Type of 3D printing material: Type of material used to manufacture the product, that depends on the 3D printing technology used. Two types of technology were used in this study: FDM, where the printer deposits fused material (different plastics) layer by layer, and stereolithography (SLA), in which a laser photo-polymerizes a resin converting it into solid material.

Quantitative variables have been described with centralization measures (mean and median); qualitative variables as numbers and percentages.

## Results

A total of 623 additive manufacturing processes have been carried out in the Orthopedic Surgery and Traumatology Department during the studied time. Research lines (Basic research, 3DP research, University collaborative projects) show the most significant activity (38.84%). Other areas of expertise that carried out a substantial number of processes were Orthopedics Oncology (21.86%) and Traumatology (20.06%). Although the number of annual processes has been similar, a different evolution by area of expertise is observed, with a larger number of processes identified in specific research lines (3DP research, University collaborative projects) in the first two years. The detailed data is shown in Table 1.

**Table 1:** Annual distribution of products by area of expertise

Area of expertise	2015	2016	2017	2018	2019	2020	Total (%)
**Spine**	–	0.64	0.64	1.44	0.48	–	**3.21**
**Upper limb**	–	–	0.80	–	0.96	–	**1.77**
**Paediatric orthopaedic**	–	0.48	–	–	–	–	**0.48**
**Basic research**	–	0.96	2.41	–	0.32	–	**3.69**
**3DP research**	0.32	9.63	10.27	4.49	0.96	0.32	**26.00**
**Foot and Ankle**	–	0.64	–	0.80	0.32	0.16	**1.93**
**University collaborative projects**	–	3.69	3.21	1.93	0.32	–	**9.15**
**Reconstructive-Infections**	–	2.89	3.85	2.57	2.41	0.16	**11.88**
**Traumatology**	–	0.16	0.96	1.28	13.80	3.85	**20.06**
**Orthopedic oncology**	–	4.82	6.10	8.03	1.28	1.61	**21.83**
**Total (%)**	**0.32**	**23.92**	**28.25**	**20.55**	**20.87**	**6.10**	**100.00**

**Fig. 2:**
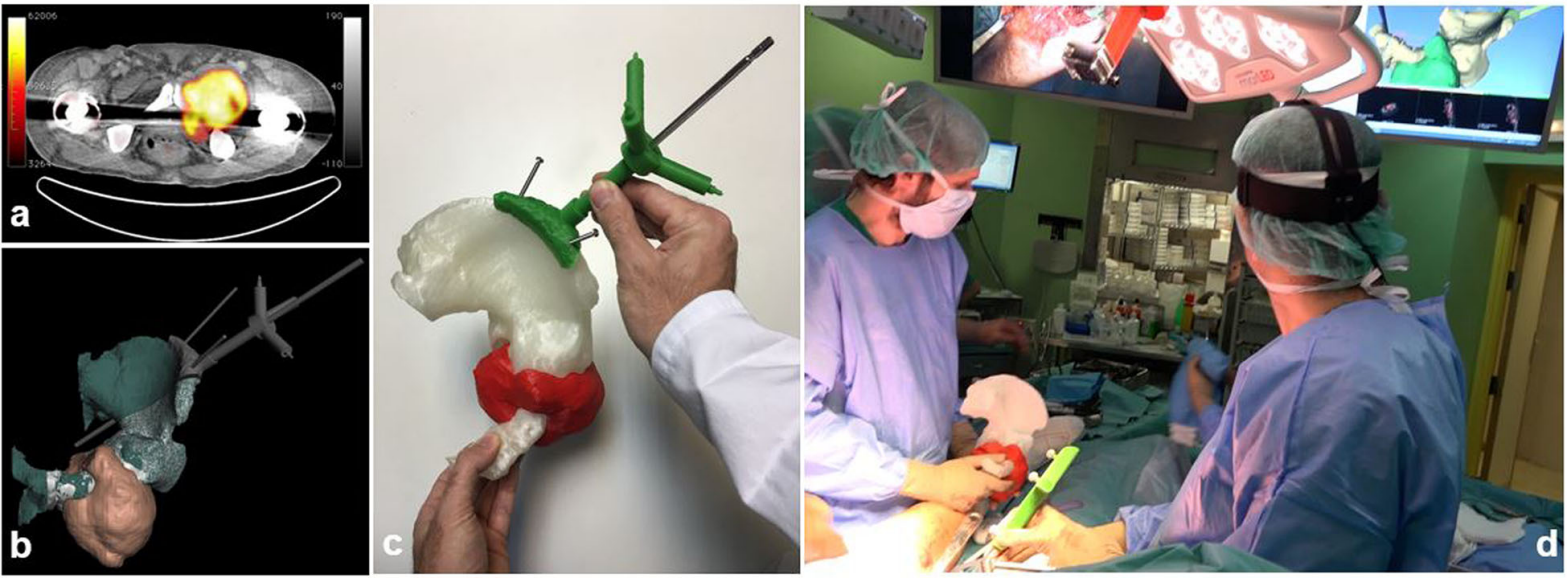
Surgical navigation of a pelvic tumor. Design and 3D Printing of patient-specific instruments. Patient radiological images **a**, 3D digital model **b**, Products [3D printed biomodel, Navigation instruments] **c**, Product utility [Intraoperative utility] **d**

The products requested were 3D printed biomodels in 87.32% of cases and surgical positioning guides in 10.75%. The remaining cases were patient-specific instruments for surgical interventions, in which 3D printing hybridization with surgical navigation or augmented reality provided added value [18, 19] (Fig. 2). The utility has been different depending on the type of product manufactured. 3D printed biomodels have been used in communication, planning, or research. On the other side, the usefulness of positioning guides or instruments has mainly been intraoperative (Table 2).

**Table 2:** Product utility

	Communication	Instrumentals	Research	Others	Pre-operative planning	Intra-Operative utility	Total (%)
**Surgical guide / Interventional procedure**	0.16	–	0.16	–	–	10.43	**10.75**
**3D Printed Biomodel**	12.36	0.48	24.40	3.21	46.39	0.48	**87.32**
**Navegation**	–	–	–	–	–	1.93	**1.93**
**Total (%)**	**12.52**	**0.48**	**24.56**	**3.21**	**46.39**	**12.84**	**100.00**

A specific analysis of areas of expertise has shown that the types of products required in each area are different (Table 3). In areas such as Traumatology, Upper Limb or Pediatric Orthopedic, 3D Printed Biomodel has been the requested product in all cases. However, Foot and Ankle, Orthopedic Oncology or Reconstructive Surgery, requested other products such as guides or patient-specific instruments in 75, 33.01, and 16.24% of cases, respectively.

**Table 3:** Distribution of products and delivery material by areas of expertise

	Spine	Upper limb	Paediatric orthopaedic	Basic research	3DP research	Foot Ankle	University collaborative projects	Reconstructive Infections	Traumatology	Orthopedic oncology	Total (%)
**Surgical guide / Interventional procedure**	**0.48**	**–**	**–**	**0.32**	**–**	**1.44**	**–**	**1.93**	**–**	**6.58**	**10.75**
MRN / DICOM	0.16	–	–	–	–	0.80	–	1.12	–	0.80	2.89
3D Model	0.32	–	–	0.16	–	0.64	–	0.48	–	5.62	7.22
Others	–	–	–	0.16	–	–	–	0.32	–	0.16	0.64
**3D Printed Biomodel**	**2.73**	**1.77**	**0.48**	**3.37**	**24.72**	**0.48**	**9.15**	**9.95**	**20.06**	**14.61**	**87.32**
MRN / DICOM	1.93	–	–	0.64	3.53	–	3.21	5.46	18.30	7.06	42.37
3D Model	0.80	1.77	0.48	1.61	16.21	0.48	3.85	4.17	1.77	7.54	36.44
Others	–	–	–	1.12	4.98	–	2.09	0.32	–	–	8.51
**Navegation**	**–**	**–**	**–**	**–**	**1.28**	**–**	**–**	**–**	**–**	**0.64**	**1.93**
MRN / DICOM	–	–	–	–	0.16	–	–	–	–	0.16	0.32
3D Model	–	–	–	–	1.12	–	–	–	–	0.48	1.61
Others	–	–	–	–	–	–	–	–	–	–	–
**Total (%)**	**3.21**	**1.77**	**0.48**	**3.69**	**26**	**1.93**	**9.15**	**11.88**	**20.06**	**21.83**	**100**

**Fig. 3:**
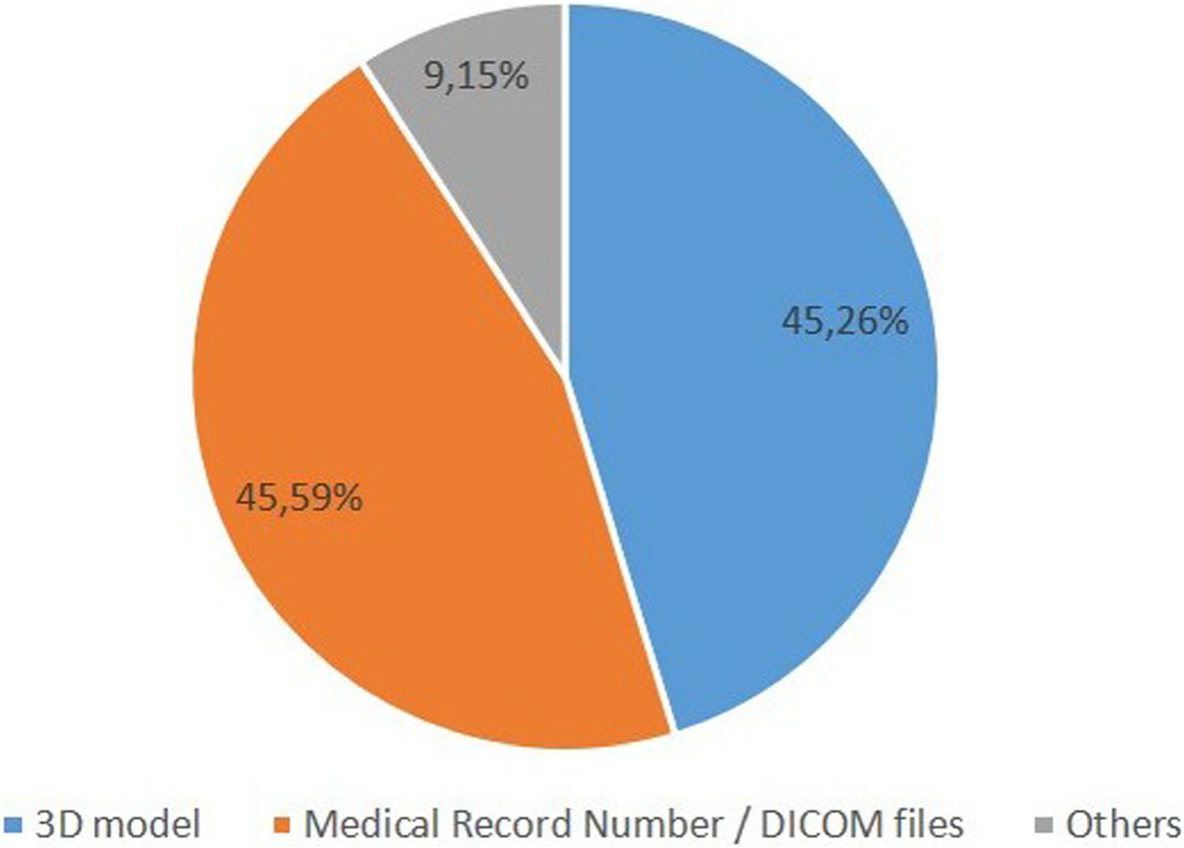
Delivery material for the manufacturing of required products

Material delivered to perform the process was MRN / DICOM (45.59%) or 3D Model (45.26%). In the remaining cases (9.15%), other materials were delivered (Fig. 3). A different percentage distribution is evidenced in delivery material according to areas of expertise and types of products required. The detailed data is shown in Table 3.

**Fig. 4:**
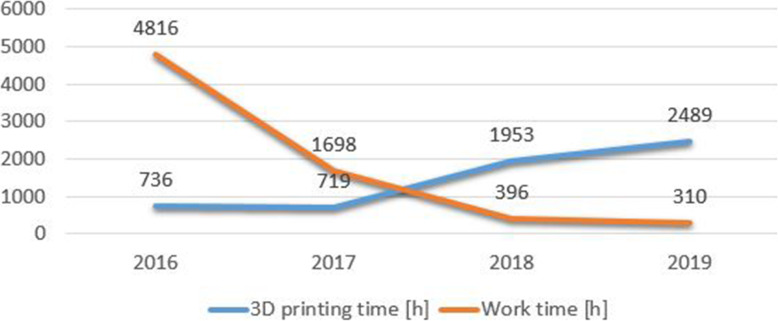
Working and 3D Printing Times. Annual distribution. Data for 2015 and 2020 are not included (Incomplete)

Total working hours were 7410, with an average of 11.89 h (median 3 h) per process. The operating time of the 3D printers was 6277 h, with an average of 10.08 h (median 5 h) per process. However, the working time and 3D printing time have been different according to the type of product required and the delivery material (Table 4). The annual distribution of working and printing hours shows two lines that cross during the study period, due to a greater number of working hours in the first years with increased printing times in recent years (Fig. 4). This evolution has been mainly due to the increasing complexity of the products that are printed, combined with the decreasing times in the segmentation and image processing necessary to obtain virtual 3D models.

**Table 4:** Technical characteristics of the projects

	Work time (hours)		3D printing time (hours)		Quantity 3D printing material (grams)	
	x	M	x	M	x	M
**Surgical guide / Interventional procedure**	**11.79**	**10**	**5,03**	**1**	**44.19**	**8**
MRN / DICOM	5.33	1	9	2,5	59.28	20
3D Model	14.84	15	2.60	1	25,98	8
Others	6.50	2.5	14.50	1	181.25	8.5
**3D Printed Biomodel**	**11.89**	**3**	**10.73**	**6**	**102.61**	**61.5**
MRN / DICOM	5.29	10	15.59	13	134.23	96.5
3D Model	20.06	2	6.46	3	76.20	33
Others	11.60	1	4.94	8	59.45	64
**Navegation**	**11.89**	**1**	**8.25**	**3.5**	**55.92**	**18**
MRN / DICOM	0.10	1	25.00	25	17.50	33
3D Model	30	0.5	4.90	3	63.60	16
Others	–	–	–	–	–	–
**Total**	**11.89**	**3**	**10.08**	**5**	**95.59**	**50**

**Fig. 5:**
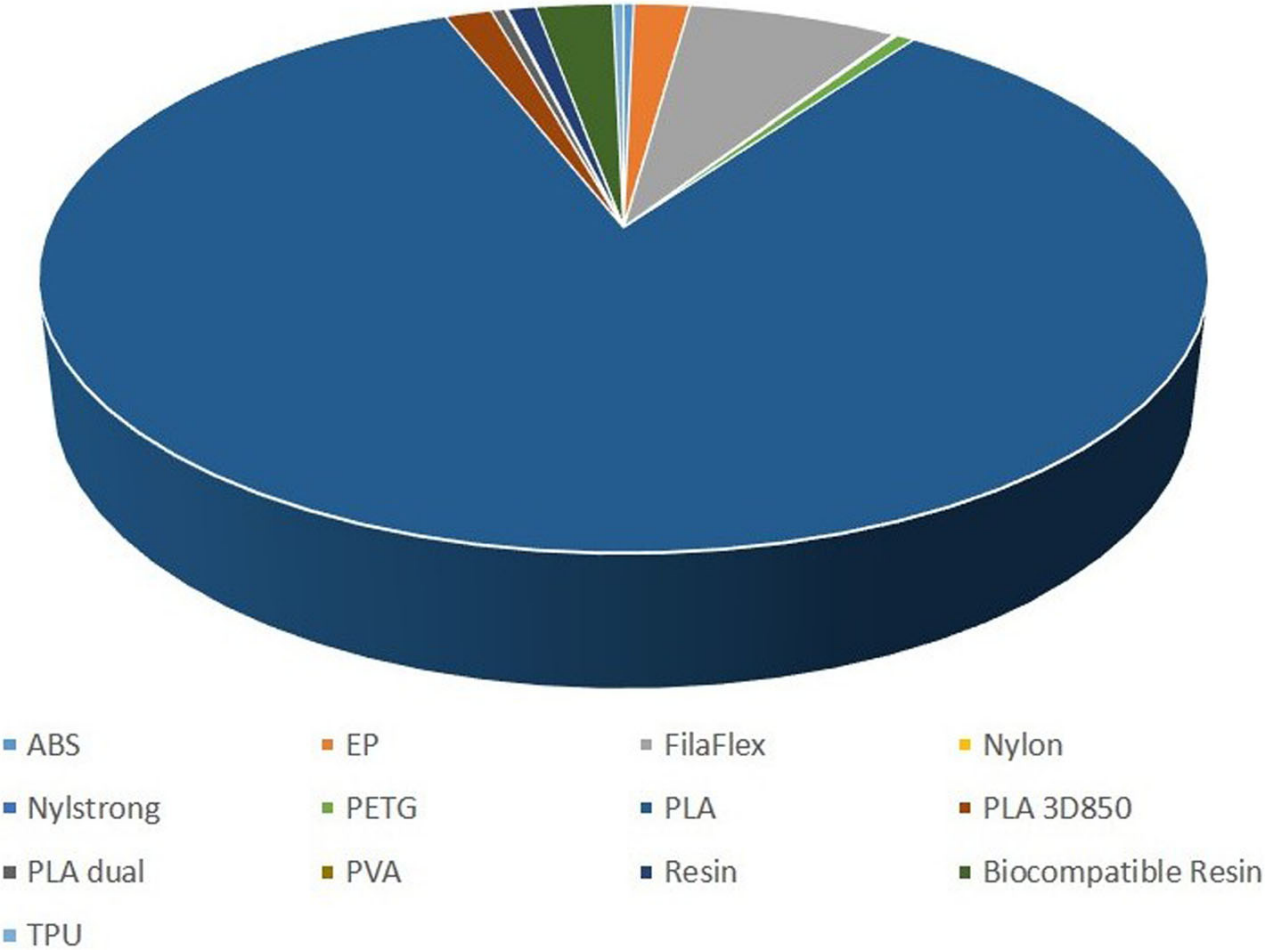
3D Printed Materials

3D printing material consumed was 59,555 g, with an average of 95.59 g (median of 50 g) per process, with 96.5% of the material used on FDM 3D printers. 3D printing material most widely used was PLA (84%). This is a rigid material that replicates bone structures with great realism. But many others have been used, either flexible such as Filaflex (thermoplastic elastomer based on polyurethane and certain additives) which allows replica of vascular structures, solid organs or muscular-tendon structures, or support materials such as PVA (polyvinyl alcohol) to optimize the post-processing and improve the quality of the printed object. The demand and production capacity of positioning guides or patient-specific instruments has led to the development of biocompatible materials, basically resins, certified for medical use that allow manufacturing these products with SLA technology. During this period, 2.29% of the products have been printed with SLA, representing 3.5% of the total material (Fig. 5).

**Fig. 6:**
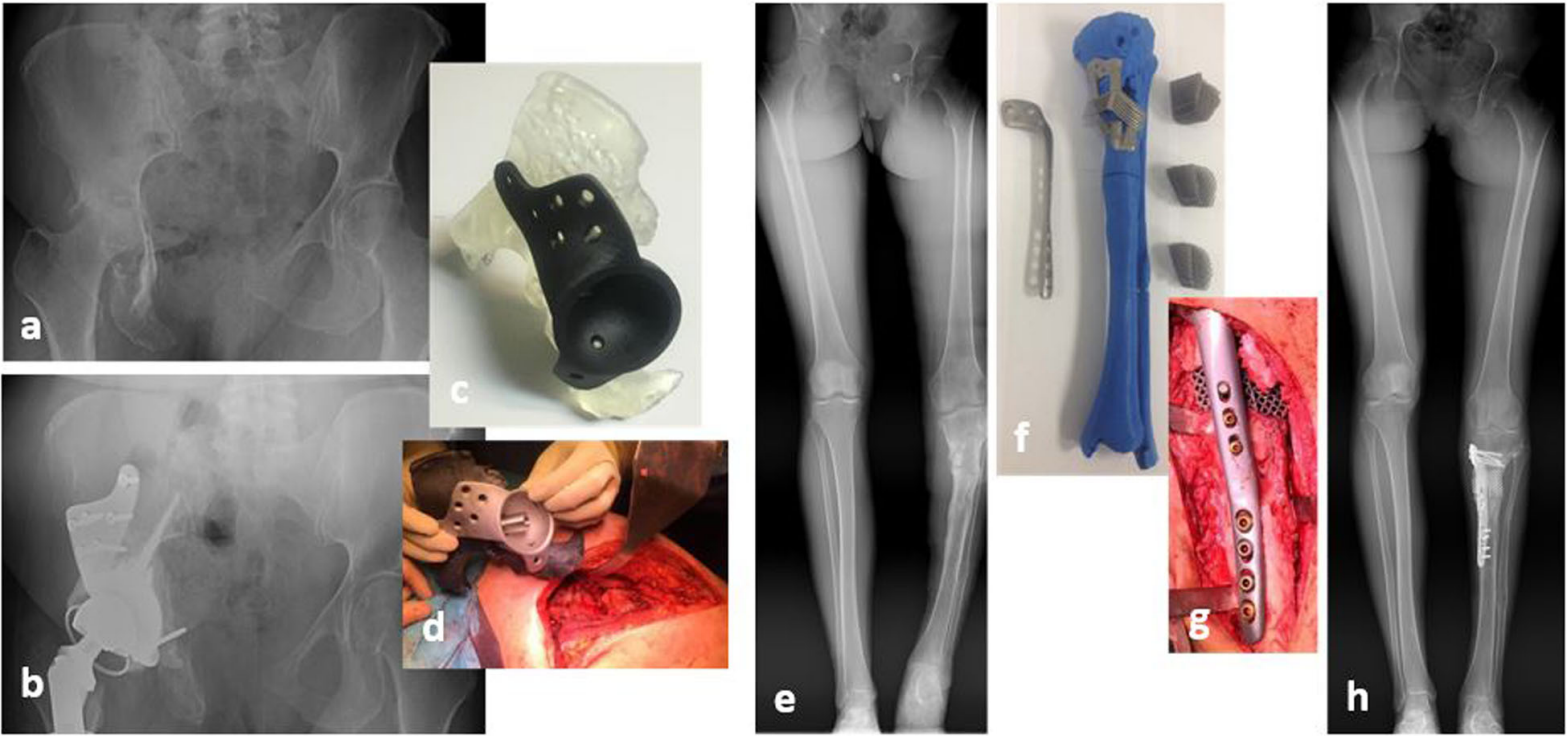
Customized implants. Pelvic implant **a-d**. Tibial implant **e-h**

The experience and accreditation as a manufacturing university hospital have made it possible to work with different companies in the sector, participating in the co-design of personalized implants [20] **(**Fig. 6**)**, and collaborating with different research groups in bone and cartilage tissue bio-impression lines [21].

## Discussion

3D technology is helping to address some of the growing complexities in healthcare, while enabling a more sustainable future as a scalable and cost-effective technology. Understood as a patient-specific process, it allows for greater efficiency throughout the entire value chain to improve results for the patient, and doing it right the first time (GIRFT methodology) through a higher level of customization and predictability [4, 22–24].

Hospital General Universitario Gregorio Marañón is a pioneer in the transversal implementation of hospital 3D printing, incorporating an “in-house” medical 3D printing laboratory integrated into the clinical workflow of more than 20 medical-surgical specialties. As a university manufacturing hospital, it is licensed to manufacture medical devices in compliance with the international standard ISO 13485 for quality management systems for medical products. This has allowed the Orthopaedic Surgery and Traumatology Department to network and coordinate production with traditional orthopedic implant manufacturers and research centers [25–30].

A university hospital includes students or doctors in training, not replacing universities but complementing training, enriching the academic environment. In the same way, a manufacturing university hospital does not replace factories. In a manufacturing university hospital, 3D printing goes hand-in-hand with translational research and teaching, acting as an accelerator for clinical innovation. 3D technology is a great tool for teaching and medical simulation, which is also carried out efficiently and in a personalized way, since training models can be manufactured that reproduce specific pathologies of real medical cases.

The integration of 3D printing into the clinical workflow has allowed complete control and monitoring of the process, from the indication to the manufacture of a customized medical-surgical solution. This adds significant value in the manufacture of guides and instruments or even customized implants, integrating 3D design as part of the therapeutic planning process, and 3D printing as part of the surgical approach [31–33].

The indicators described in this study allow evaluation and proposal of specific corrective actions according to the results obtained. Annual production by areas of expertise has changed during the study period. Identification and optimization of specific software and hardware, materials or manufacturing parameters have been research objectives, which has required not only implementation of a high number of processes in first two years, but also an increase in working time of technical and medical staff assigned to the achievement of these processes.

It is important to identify the areas of expertise that have the greatest potential for the integration of 3D printing technology. The Radiological Society of North America 3D printing group (3D Special Interest Group RSNA) has reviewed and classified the clinical cases in which it is more efficient to use 3D printed biomodels, and has concluded that in simple fractures the role of 3D printing is not as useful as in complex fractures, hip dysplasia or bone tumours with joint involvement [34]. In our study, *Reconstructive Surgery*, which included deformities, degenerative joint pathology, infections or arthroplasties, *Traumatology*, which managed fractures of any anatomical location or age of presentation, and *Orthopedic Oncology*, represented 53.77% of the global activity. If we take into account that 38.84% was research activity, the remaining areas of expertise accounted for 7.39% (46 cases) of the total activity. With these findings, it is important to highlight the role of the manufacturing university hospital allowing the adaptation and optimization of response times, of great relevance in areas such as Traumatology or Orthopedic Oncology, where traditional manufacturing presents restrictions such as process outsourcing or associated costs.

The utility of 3D printed biomodels for preoperative planning has been of great interest in recent years [35]. In our study, 87.32% of the required products were 3D printed biomodels used not only for surgical planning but also for communication or research.

The availability of machines for “in-house” manufacturing by means of FDM or SLA technologies has allowed the production of 3D Printed Biomodels, Surgical Guides and Patient-specific Instruments, and the collaborative work with manufacturing companies has facilitated the co-design and production of patient-specific implants. In this way, complete traceability can be maintained over each stage of creation without interrupting the workflow. It allows attending in times of very tight therapeutic windows and with the solvency of a multidisciplinary team that accumulates valuable experience and knowledge of the patient that would otherwise remain fragmented. This is enabled by the point-of-care manufacturing model. It is also in line with the regulatory framework of this technology applied to personalized medicine, which identifies the prescribing physician as the final responsable for the process, including the design of the custom-made product [36–39].

## Conclusions

This study identifies the possibilities of integrating 3D printing technology in a Department of Orthopedic Surgery and Traumatology. This experience allowed us to identify the conceptual evolution of the hospital workflow in additive manufacturing processes from “Do it yourself” to “POCM”. A descriptive monocentric study makes it difficult to extrapolate the results, so it is essential to propose specific multicenter studies and consensus documents.

## Data Availability

The authors declare that they have followed their centre’s protocols on the publication of patient data. All data analyzed during the current study are available from the corresponding author on reasonable request.

## Abbreviations

3D: Three dimensional
3DP: Three dimensional printing
COVID-19: Coronavirus disease 2019
DICOM: digital imaging and communication on medicine
FDM: Fused deposition modeling
GIRFT: Getting It Right First Time
ISO: International Organization for Standardization
MRN: medical record number
PLA: polylactic acid
POC: point of care
POCM: point of care manufacturing
PVA: polyvinyl alcohol
SLA: Stereolithography
UPAM3D: Advanced Planning and 3D Manufacturing Unit
